# Delirious Mania: Can We Get Away with This Concept? A Case Report and Review of the Literature

**DOI:** 10.1155/2012/720354

**Published:** 2012-11-12

**Authors:** Rajshekhar Bipeta, Majeed A. Khan

**Affiliations:** ^1^City Nursing Home, Himayath Nagar, Hyderabad 500029, Andhra Pradesh, India; ^2^Deccan College of Medical Sciences, Hyderabad 500058, Andhra Pradesh, India

## Abstract

*Background*. Delirious mania (DM) as a clinical entity is well described, yet is often unrecognized in clinical practice. While most often misdiagnosed as acute psychotic episodes of organic delirium, these patients meet the criteria for mania with attendant delirium and pose therapeutic challenges. In addition to the case presentation, this paper also discusses the available literature on DM. *Case Presentation*. A 29-year-old man with DM was treated with a combination of electroconvulsive therapy (ECT), divalproex 2000 mg/day, loxapine 100 mg/day, and lorazepam 4 mg/day. He demonstrated clinically significant improvement by day 10, which persisted through the twelve-month follow-up period. *Conclusions*. DM is a severe psychiatric syndrome which should be accurately diagnosed. Patients with DM should be treated aggressively, especially with ECT. Lack of recognition of DM can lead to serious morbidity or fatal outcomes. Though the concept of DM is well established, recent psychiatric literature does not make a mention of this life threatening yet treatable condition. We propose that there is a dire need to keep this concept alive.

## 1. Introduction

Bipolar disorder (BD) is classically viewed as a condition characterized by periods of euphoric excitement and depressive retardation, which is easy to diagnose and treat, whose treatment is exclusively pharmacological, and whose outcome is generally favourable. However, recent advances have shown that the rubric “BD” actually encompasses a variety of conditions. Also, it may have comorbidity with other psychiatric conditions. The diagnosis of BD becomes difficult when there is a variation of the classical picture. Treatment efficacy depends on the type of bipolar state. Several pharmacological agents have been introduced; the choice of the most appropriate drug in the individual patient has become much more complex. 

Delirious mania (DM), also known as Bell's mania [1], is characterized by excitement, grandiosity, emotional lability, psychosis and insomnia characteristic of mania, altered consciousness, and disorientation characteristic of delirium [2–6]. The term DM was coined by Kraeplin, but was initially described by Calmeil [7], and reported to have a high morbidity [8] and mortality [1, 9]. Bell, in 1849, reported 40 patients out of 1700 admissions, who had features suggestive of DM and 75% of these patients subsequently died [1]. 

DM is associated with BD, and its symptoms encompass mania and acute mental confusion [6, 10, 11]. 5–20% of all patients with acute mania show signs of delirium [2]. Klerman proposed staging of manic spectrum as follows: normal, neurotic, hypomanic, manic, and delirious [12]. DM has marked similarity to stage III mania as described by Carlson and Goodwin [13]. The transition of mania to DM is marked by emergence of confusion, more hallucinations, and a marked intensification of the manic symptoms. A dreamlike clouding of consciousness may occur [14]. 

According to Dunayevich and Keck [15], symptoms in DM are similar to schizophrenia [13], with presence of severe anxiety, frenzied activity, and incoherence [15]. Almost all patients with DM exhibit signs of catatonia [2, 3, 16]. Taylor and Fink theorised DM as a form of catatonia because of the presence of catatonic features, and good response to electroconvulsive therapy ECT [2, 17].

Many cases are precipitated by medical or neurological conditions [11] or by psychoactive substances [2, 11]. Adolescents and children are particularly prone to the very rapid development of DM [14]. 

DM was initially thought to be an uncommon syndrome, as many cases used to go unrecognized. Though the concept of DM is now documented in literature, not much is known about its aetiopathogenesis [18] and core clinical features. There are no treatment guidelines. We did pubmed and google scholar search for the available literature regarding DM. The published literature mostly consists of case series [19]. Various controversies exist regarding proper nomenclature. Various terms such as excited catatonia [18], lethal catatonia, and malignant catatonia, along with the popular term “DM” exist (2). 

Carlson and Goodwin did a longitudinal study of 20 patients with mania. Six (33%) patients developed disorientation suggesting that DM is a frequent occurrence [13]. 

In 1996, Fox and Bostwick reported the case of a man with DM who failed to respond to standard antimanic treatment. In view of the deterioration of his condition, he was finally sedated with propofol, which enabled treatment of his uncontrolled life-threatening manic state. The authors concluded that while propofol would not be a practical first-line treatment for agitated psychiatric patients, it may be helpful for refractory cases [20]. 

In 2001, Weintraub and Lippmann reported two cases of elderly patients with mania whose initial presentation was delirium. Both patients responded well to divalproex (mood stabilizer). The authors concluded that mania should be a differential diagnosis of elderly patients who present with confusion, disorientation, and perceptual disturbances, especially if there is a history of bipolar disorder [21].

Karmacharya et al. suggested that the definitive treatment for DM is ECT. In the absence of ECT, they advised use of high-dose benzodiazepines. They opined that clozapine, quetiapine, lithium, and valproate cannot be considered first-line treatments in view of a reasonably delayed onset of action. They also cautioned against the use of typical antipsychotics and anticholinergics [19]. 

In 2009, Nicolato et al. reported successful treatment of DM with a combination of olanzapine and ECT. However, they too cautioned against the use of antipsychotics including the atypical, as they may cause neuroleptic malignant syndrome (NMS), or even aggravate the catatonia, especially the malignant catatonia [11]. 

Barahona-Corrêa et al. reviewed all cases with mania, hypomania, or mixed affective state from 2006 to 2007. Those with delirious features and a diagnosis of DM were specifically reviewed. 14% of their patients had medically unexplained delirium. Four (out of 14 patients) had a final diagnosis of DM, and in three of them, DM occurred during manic/mixed affective states. Compared to non-DM cases, DM cases had longer inpatient stay, acute onset of symptoms, hyperthermia, catatonia, autonomic instability, sleep disturbances, coprolalia, and persistence of delirium for over a week. ECT was preferred in such cases. The authors concluded that DM occurs rarely in BD, has typical clinical features, with a tendency for recurrence [22]. 

The most recent work on DM is by Lee et al. in 2012. They comment that DM does not find mention in the current nosology as a separate diagnosis. The authors present five cases of DM who had “concurrent manic and delirious symptoms during hospitalization, and medical workup failed to uncover an organic cause for either mania or delirium.” They caution that in view of high medical comorbidity in BD patients, there is a risk of missing DM. The authors conclude that BD patients are at increased risk of delirium, with a tendency for recurrence. They recommend early recognition and aggressive management, particularly with ECT [23].

We describe a case of DM that warranted aggressive management because of extreme agitation and exhaustion. To our knowledge, this is the first case of DM in published literature, where standardized instruments were used to track symptoms longitudinally. 

## 2. Case Presentation

### 2.1. Sociodemographic and Clinical Details

Our patient was a 29-year-old, unmarried, unemployed graduate man from middle socioeconomic status and urban Asian background. He had a two-year history of episodic illness with interepisodic premorbid level of functioning. Earlier two episodes of mania of very high severity resolved rapidly, with treatment. When normal, he used to stop medication. He was completely off psychotropic medication for six months before the current episode. 

### 2.2. History of Present Illness

He presented with an acute onset, third episode, of ten-day duration, characterized by assaultive behaviour, incessant and boastful talk, excessive planning, overfamiliarity, increased sexual desire, and decreased need for sleep and food. He was continuously reciting from the holy books; his relatives saw no coherence in it; it was totally misplaced. He engaged in continuous charity, without any signs of stopping it. The symptoms increased in severity within the previous six hours. 

Along with these symptoms, for previous ten days, our patient was confused, had forgetfulness for recent events, and was not able to consistently identify even his close family members. He was seeing images of snakes on fans and would become extremely fearful. He was sometimes urinating and defecating inside the house, and in his clothes.

### 2.3. Physical Examination

Physical examination showed elevated blood pressure (BP) (170/110 mm Hg), increased heart rate (pulse 102/minute), increased respiratory rate (24/minute), and a body weight of 102 kg. The temperature was 98.6 degrees F. Otherwise, the findings in the physical examination, including a detailed neurological assessment, were unremarkable. He was exhausted; however, there were no signs of dehydration.

### 2.4. Mental Status Examination

Rapport could not be established. He was extremely agitated, cursing everybody, including the doctors, and spitting at others. He even assaulted the hospital staff. He was turning everything upside-down, pushing the hospital furniture, jumping on the bed, dragging the mattresses, destroying all that which was in his reach. He had to be tied securely to the cot, but was even pulling the cot. He was making sexual advances towards fellow patients and hospital staff. These prolonged periods of extreme agitation would be followed by brief periods of sudden calmness and muteness.

He was continuously talking, loudly, some meaningless and mostly incoherent words. There was echolalia, echopraxia, stereotypy, word salad, flight of ideas, and clang associations. He was having delusions of grandiosity with mood congruent delusions of persecution and reference. He believed that he was God's messenger, with a mission to eradicate suffering and could communicate with others by telepathy. He strongly suspected that his neighbours were discussing his greatness. He had second person auditory hallucinations and would hear threatening voices of devils, which were envious of his power and wanted to kill him by sending secret executioners. He had visual hallucinations and could see images of snakes sent to execute him. He believed that the treating psychiatrists were butchers sent by persecutors to finish him off. He demonstrated extreme fluctuations of mood, ranging from extreme dysphoria to infectious jocularity. He would suddenly burst into tears. He was confused and had fluctuating levels of consciousness. He was inattentive, disoriented in time, could not recognize his family members, and said that he was in a railway station. His insight into his illness was impaired.

### 2.5. Other Details

Our patient was premorbidly well adjusted. There was no history of medical (including hypertension and other cardiovascular disorders) or neurological disorders, or substance abuse. There was no history to suggest infection in recent past. His elder sister was suffering from bipolar disorder, but was never treated. 

### 2.6. Investigations

Laboratory studies, including complete blood picture, renal functions, blood sugars, liver and thyroid functions, urine analysis, chest X ray, and electro-cardiogram did not reveal any abnormalities. A brain computed tomography and serum creatinine phospho kinase (CPK) estimation could not be done as the family members were unwilling because of financial constraints. 

### 2.7. Diagnosis

He was diagnosed to have “bipolar disorder I, most recent episodemanic, severe withpsychotic features” as per Diagnostic and Statistical Manual of Mental Disorders (DSM IV criteria) [24]. He had associated features suggestive of delirium. 

### 2.8. Assessments

At various time points, different instruments were applied to measure the illness severity and treatment response (Table 1). The Young's Mania Rating Scale (YMRS) is an 11-item, clinician-administered scale to measure the severity of mania. Higher scores reflect more severe psychopathology [25]. The Clinical Global Impression-Severity scale (CGI-S) is a 7-point scale that requires the clinician to rate the severity relative to experience with patients with similar diagnosis. The Clinical Global Impression-Improvement scale (CGI-I) is a 7-point scale that assesses change compared to baseline. The Clinical Global Impression-Efficacy Index (CGI-E) is a 4 × 4 point scale that assesses the therapeutic effect vis-a-vis emergence of side effects [26]. Mini Mental Status Examination (MMSE) is used to assess the severity of cognitive impairment and to document an individual's response to treatment [27]. To get an objective evidence of delirium, we administered MMSE at baseline (day 1) and also at day 12 (discharge). However, because of extreme aggression, the patient did not respond to all the items at baseline. At day 12, he did cooperate for the same. Hence, Table 1 mentions MMSE scores only at day 12, and not at baseline.

### 2.9. Treatment Details and Course in the Hospital

**Figure 1:**
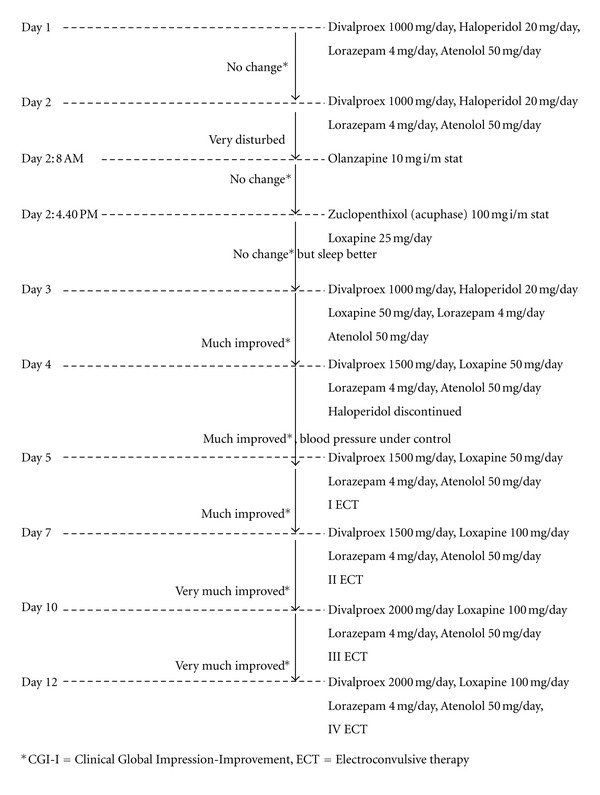
Flowchart depicting the treatment details in our patient.

On admission, our patient was “extremely ill.” ECT was not started as he had high BP, instead he was started on pharmacological management (Figure 1). 

The elevated BP was managed with atenolol 50 mg/day. He was started on divalproex 1000 mg/day, haloperidol 20 mg/day, and lorazepam 4 mg/day. On day 2, as he was still very disturbed, olanzapine 10 mg i/m was administered. As there was no relief even after 8 hours, zuclopenthixol (Acuphase) 100 mg i/m was given, and loxapine (an antipsychotic drug) 25 mg/day was added.

By day 3, there was no relief; however, sleep was better. Loxapine was increased to 50 mg/day. By day 4, the aggression was better and clinically he was “much improved.” He was now sleeping from 6 to 7 hours per night. Divalproex was increased to 1500 mg/day, and haloperidol was discontinued.

By day 5, as the BP was under control (130/90 mm Hg), bilateral ECT was started. The patient was maintaining the same improvement at day 7. He would quietly lie down, paranoia was coming down and he started accepting food and water. He was now amenable to suggestions and started calling others with respect. Loxapine was increased to 100 mg/day. By day 10, the patient had shown “marked improvement” and there was no evidence of any psychopathology. His self-care improved. His insight was relatively improved, the consciousness was clear and he was well oriented. Divalproex was increased to 2000 mg/day.

A total of four sessions of ECT were administered. On day 12 (day of discharge), there was “marked improvement with no side effects.” He did not remember what had happened till one day back, for the previous nineteen days. Though we could not administer MMSE in detail on admission, his score on discharge was 28/30 (normal range). He was discharged on divalproex 2000 mg/day, loxapine 100 mg/day, lorazepam 4 mg/day, and atenolol 50 mg/day. He was referred to physician for further management of hypertension.

### 2.10. Follow-Up Period

About two weeks after-discharge, lorazepam was tapered off, and he was maintained on divalproex 2000 mg/day and loxapine 100 mg/day. He maintained euthymia for the next one year and then was lost to follow up.

## 3. Discussion

This could well be our “most severe” recorded case of mania, who responded wonderfully to treatment. A classical case of bipolar-I, but with an element of delirium. A case of DM as per Bond's criteria, (i) acute onset of symptoms, (ii) presence of mania, (iii) features of delirium, (iv) history of mania, (v) family history of bipolar disorder, and (vi) responsivity to treatment for mania [28]. He also met Fink's criteria for DM and catatonia (Table 2) [3, 10]. 

NMS was the differential diagnosis considered. The classic features of NMS include muscular rigidity, altered sensorium, autonomic instability, and hyperthermia (i.e., temperature >100.4°F). Associated features include akinesia, mutism, obtundation, and agitation. The serum CPK level is increased in nearly all cases [29]. But NMS was ruled out in our patient as (i) the neurological examination was within normal limits. There was no muscular rigidity, (ii) there was no fever, and (iii) our patient was off all psychotropic medication since six months before the current episode. However, serum CPK level could not be ascertained in our patient.

Discussing the relationship between “psychogenic”/“functional,” and “organic” in delirium, Hart talks about the confusion that has resulted from the diversities of meaning with which both those terms are used. He suggests that the term “psychogenic” indicates a “mode of explanation.” It does not imply that “those causal processes are incapable of being conceived in neurological terms…” [30]. Swartz et al. cautioned that “the recognition of DM is critical once an “organic aetiology” has been excluded. Not treating such patients effectively and incorrect attribution to drugs can be dangerous” [31]. 

Early recognition of sleep disturbance is crucial for the prevention of relapse or recurrence in BD patients [32]. A point worth mentioning is that our patient's sleep was disturbed since ten days before admission, but exacerbation to this height of mania reached within hours. After starting loxapine, the sleep started improving day 3 onwards. Loxapineis a serotonin-dopamine antagonist and a member of the dibenzoxazepine class [33]. Several researchers argue that loxapine may behave as anatypical antipsychotic [34]. Typical antipsychotics are found to be beneficial in delirium [35]; however, some authors caution against their usage in DM [19, 36]. Atypical antipsychotics are proposed to be useful in DM [19, 23, 37, 38]; but, some researchers consider these to be detrimental in presence of catatonic features [23, 38].

In our patient, a brief course of four bilateral ECTs was further able to hasten the recovery. This is in keeping with earlier reports [2, 8, 19, 39, 40], where patients had speedy response with few sessions of ECT. ECT should be considered in early stages of DM [40, 41], as first-line treatment [28] and lifesaving [42, 43]. 

GABA-ergic transmission in the orbitofrontal premotor and motor cortices is implicated in the aetiopathogenesis of catatonia, hence, GABA-A potentiators like high-dose lorazepam (3-4 mg/day) may benefit some patients [19, 44]. However, our patient was treated with lorazepam 4 mg/day from the very first day, with no appreciable benefit [23]. Catatonia may also be attributed to glutamate (NMDA receptor) hyperactivity. Therefore, in case of nonresponse with lorazepam or ECT, glutamate antagonist therapy with amantadine may help [45]. 

Our patient had a very brief inpatient stay, just 12 days, and this is in contrast to earlier case reports needing prolonged hospitalization [22]. One controversy related to our case may be that we changed treatment drastically, and the drugs were given at high doses. But, we should remember that DM is a life-threatening condition and should be treated aggressively. In Kraines' word “one seems justified in utilizing just as much medication as necessary, no matter what the standard dosage is, in order to procure sufficient quiet to prevent cardiac failure.... The prognosis, which is considered so ominous, might be changed under such treatment” [9]. 

## 4. Conclusions

Our case illustrates the real world challenges which clinicians face in their day-to-day practice. Though case reports find the lowest position in evidence-based research, these continue to guide researchers in planning more rigorous methodologically sound studies.

Extreme excitement during the manic phase of BD should alert to the possibility of delirium. DM, an extremely severe, yet rare condition [1], involves severe incessant agitation, leading to a medical emergency. There is a high likelihood to misdiagnose these cases as organic mania. Lack of recognition of this condition may lead to mismanagement of the course of illness [28]. Such patients need aggressive management, especially with ECT. 

Though this condition was first described about one hundred and eighty years back, and there is good literature in this regard, it is unfortunate that there is no mention of this life threatening, yet treatable condition in recent systems of classification and text books of psychiatry. DM should be considered a subtype of catatonia [3, 16, 17, 23].

The fact remains that there is a separate entity called DM, which needs specialized attention, and we cannot get away with this useful concept.

## Figures and Tables

**Table 1:** Assessments at various time points in our patient.

Scales/scores	Day 1	Day 4	Day 7	Day 10	Day 12
YMRS (0–60)	58	33	19	10	7
CGI-S (1–7)	7, extremely ill	4, moderately ill	3, mildly ill	1, normal	1
CGI-I (1–7)	NA	2, much improved	2	1, very much improved	1
CGI-E (01–16)	NA	05, moderate therapeutic effect. No side effects	05	01, marked therapeutic effect. No side effects	01
MMSE (1–30)	—	—	—	—	28

YMRS: Young's Mania Rating Scale, CGI: Clinical Global Impression, CGI-S: CGI-Severity scale, CGI-I: CGI-Improvement scale, MMSE: Mini Mental Status Examination, NA: Not Applicable.

**Table 2:** Catatonia subgroups: symptoms of delirious mania and catatonia [3, 10].

Delirious mania	Catatonia*
Present	

Sudden onset/intense excitement	
Stereotypy	
Tachycardia	
Tachypnoea	
Hypertension	
Pressured speech/mutism	

Present	Present

Disorganized thoughts Disorganized speech Refusing food and fluids	Grandiosity
	Emotional lability
	Delusions
	Insomnia
	Disorientation
	Altered consciousness
	Negativism
	Flight of ideas

Absent	

Hyperthermia	
Posturing	

Absent	

Catalepsy	
Rigidity	
Cycle from excited state to stuporous state	

^∗^Requires 2 or more signs for ≥24 hours [3].

## References

[1] Bell L (1849). On a form of disease resembling some advanced stage of mania and fever. *The American Journal of Insanity*.

[2] Fink M (1999). Delirious mania. *Bipolar Disorders*.

[3] Detweiler MB, Mehra A, Rowell T, Kim KY, Bader G (2009). Delirious mania and malignant catatonia: a report of 3 cases and review. *Psychiatric Quarterly*.

[4] Akiskal HS, Kaplan HI, Sadock BJ (1995). Mood disorders: clinical features. *Comprehensive Textbook of Psychiatry*.

[5] Akiskal HS, Maj M, Akiskal HS, Lopez-Ibor JJ, Sartorius N (2002). Classification, diagnosis and boundaries of bipolar disorders. *Bipolar Disorder*.

[6] Jung WY, Lee BD (2010). Quetiapine treatment for delirious Mania in a military soldier. *Primary Care Companion to the Journal of Clinical Psychiatry*.

[7] Calmeil LF (1832). *Dictionnaire de Medicine: Our repertoire general des sciences medicales considerees sous le rapport theorique et pratique*.

[8] Friedman RS, Mufson MJ, Eisenberg TD, Patel MR, Kahn MW (2003). Medically and psychiatrically ill: the challenge of delirious mania. *Harvard Review of Psychiatry*.

[9] Kraines SH (1934). Bell’s mania. *The American Journal of Psychiatry*.

[10] Fink M, Taylor MA (2001). The many varieties of catatonia. *European Archives of Psychiatry and Clinical Neuroscience*.

[11] Nicolato R, Costa-Val A, Souza A, Salgado JV, Teixeira AL (2009). Delirious mania associated with bipolar disease in a Brazilian patient: response to ECT and olanzapine. *Journal of Neuropsychiatry and Clinical Neurosciences*.

[12] Klerman GL (1981). The spectrum of mania. *Comprehensive Psychiatry*.

[13] Carlson GA, Goodwin FK (1973). The stages of mania. A longitudinal analysis of the manic episode. *Archives of General Psychiatry*.

[14] Moore and Jefferson (2004). Bipolar disorder (DSM-IV-TR #296. 0-296. 89). *Handbook of Medical Psychiatry*.

[15] Dunayevich E, Keck PE (2000). Prevalence and description of psychotic features in bipolar mania. *Current Psychiatry Reports*.

[16] Fink M, Taylor MA (2006). Catatonia: subtype or syndrome in DSM?. *The American Journal of Psychiatry*.

[17] Taylor MA, Fink M (2003). Catatonia in psychiatric classification: a home of its own. *The American Journal of Psychiatry*.

[18] Pruett JR, Rizvi ST (2005). A 16-year-old girl with excited catatonia treated with low-dose oral lorazepam. *Journal of Child and Adolescent Psychopharmacology*.

[19] Karmacharya R, England ML, Öngür D (2008). Delirious mania: clinical features and treatment response. *Journal of Affective Disorders*.

[20] Fox FL, Bostwick JM (1997). Propofol sedation of refractory delirious mania. *Psychosomatics*.

[21] Weintraub D, Lippmann S (2001). Delirious mania in the elderly. *International Journal of Geriatric Psychiatry*.

[22] Barahona-Corrêa B, Fernandes J, Alves da Silva J, Neto B, Almeida J (2010). P01-08—mania, mania with delirium and delirious mania. *European Psychiatry*.

[23] Lee BS, Huang SS, Hsu WY, Chiu NY (2012). Clinical features of delirious mania: a series of five cases and a brief literature review. *BMC Psychiatry*.

[24] American Psychiatric Association (1994). *Diagnostic and Statistical Manual of Mental Disorders*.

[25] Young RC, Biggs JT, Ziegler VE, Meyer DA (1978). A rating scale for mania: reliability, validity and sensitivity. *British Journal of Psychiatry*.

[26] Guy W (1976). *ECDEU Assessment Manual for Psychopharmacology*.

[27] Folstein MF, Folstein SE, McHugh PR (1975). ‘Mini mental state’. A practical method for grading the cognitive state of patients for the clinician. *Journal of Psychiatric Research*.

[28] Bond TC (1980). Recognition of acute delirious mania. *Archives of General Psychiatry*.

[29] Kohen D, Bristow M (1996). Neuroleptic malignant syndrome. *Advances in Psychiatric Treatment*.

[30] Hart B (1936). Delirious mania. *British Medical Journal*.

[31] Swartz MS, Henschen GM, Cavenar JO, Hammett EB (1982). A case of intermittent delirious mania. *The American Journal of Psychiatry*.

[32] Brambilla C, Gavinelli C, Delmonte D (2012). Seasonality and sleep: a clinical study on euthymic mood disorder patients. *Depression Research and Treatment*.

[33] Stahl SM (2000). *Essential Psychopharmacology: Neuroscientific Basis and Practical Applications*.

[34] Glazer WM (1999). Does loxapine have “atypical” properties? Clinical evidence. *Journal of Clinical Psychiatry*.

[35] Alici-Evcimen Y, Breitbart W (2008). An update on the use of antipsychotics in the treatment of delirium. *Palliative and Supportive Care*.

[36] Mann SC, Caroff SN, Bleier HR, Welz WK, Kling MA, Hayashida M (1986). Lethal catatonia. *The American Journal of Psychiatry*.

[37] Loo C, Katalinic N, Mitchell PB, Greenberg B (2011). Physical treatments for bipolar disorder: a review of electroconvulsive therapy, stereotactic surgery and other brain stimulation techniques. *Journal of Affective Disorders*.

[38] Fricchione G, Bush G, Fozdar M, Francis A, Fink M (1997). Recognition and treatment of the catatonic syndrome. *Journal of Intensive Care Medicine*.

[39] Strömgren LS (1997). ECT in acute delirium and related clinical states. *Convulsive Therapy*.

[40] Danivas V, Behere RV, Varambally S, Rao NP, Venkatasubramanian G, Gangadhar BN (2010). Electroconvulsive therapy in the treatment of delirious mania: a report of 2 patients. *Journal of ECT*.

[41] Jarvie HF, Hood MC (1952). Acute delirious mania. *The American Journal of Psychiatry*.

[42] Rudorfer MV, Henry ME, Sackeim HA, A, Kay J, Lieberman JA (2003). Electroconvulsive therapy. *Psychiatry*.

[43] Vasudev K, Grunze H What works for delirious catatonic mania?.

[44] Northoff G (2002). Catatonia and neuroleptic malignant syndrome: psychopathology and pathophysiology. *Journal of Neural Transmission*.

[45] Carroll BT, Goforth HW, Thomas C (2007). Review of adjunctive glutamate antagonist therapy in the treatment of catatonic syndromes. *Journal of Neuropsychiatry and Clinical Neurosciences*.

